# Urban health performance configurations in Chinese cities: longitudinal fsQCA, 2019–2024

**DOI:** 10.3389/fpubh.2026.1956149

**Published:** 2026-09-14

**Authors:** Donghai Ye, Juan Wang

**Affiliations:** 1 School of Physical Education, Weifang University, Weifang, Shandong, China; 2 School of Physical Education and Health, Zhaoqing University, Zhaoqing, Guangdong, China

**Keywords:** sustainable urban health, city sustainability, fuzzy-set qualitative comparative analysis, longitudinal configurations, China.

## Abstract

**Introduction:**

Composite city indices can rank urban health but rarely explain which conjunctions of public provision, economic capacity, resident action, and environmental conditions are sufficient, whether those conjunctions persist, or how cities move between them over time. This study addresses this configurational and temporal gap using longitudinal evidence from Chinese cities.

**Methods:**

We analyzed a balanced panel of 296 Chinese cities from 2019 to 2024, comprising 1,776 city-year observations. Urban health utility (HR), derived from the Tsinghua Urban Health Index (TUHI), was used as the outcome, while government health services (HS), health infrastructure (HI), health industry (HP), health behavior (HA), and health environment (HE) were treated as conditions. Longitudinal fuzzy-set qualitative comparative analysis (fsQCA) was conducted using pooled calibration anchors, supplemented by annual models, pooled/between-year/within-city diagnostics, dominant-configuration transitions, robustness analyses, and a 1-year lag model.

**Results:**

No single condition met the necessity-consistency threshold of 0.90. Two sufficient configurations for high HR were identified: ~HS^*^HI^*^HA^*^~HE (consistency = 0.926; raw coverage = 0.329) and HS^*^HI^*^HP^*^HA (consistency = 0.877; raw coverage = 0.476). Their union had a consistency of 0.872 and coverage of 0.540. The four-domain HS^*^HI^*^HP^*^HA configuration recurred from 2021 to 2024 and was reproduced unchanged in the lagged model. Sample perturbations generally retained the main configurations, whereas compressed calibration anchors and subgroup analyses revealed sensitivity and heterogeneity.

**Discussion:**

High urban health performance is associated with multiple sufficient configurations rather than a single necessary condition. The longitudinal strategy distinguishes recurring configurational relations from changing city membership over time and supports differentiated policy portfolios while retaining environmental safeguards. These findings represent set-theoretic associations rather than causal effects.

## Graphical Abstract

**Figure:**
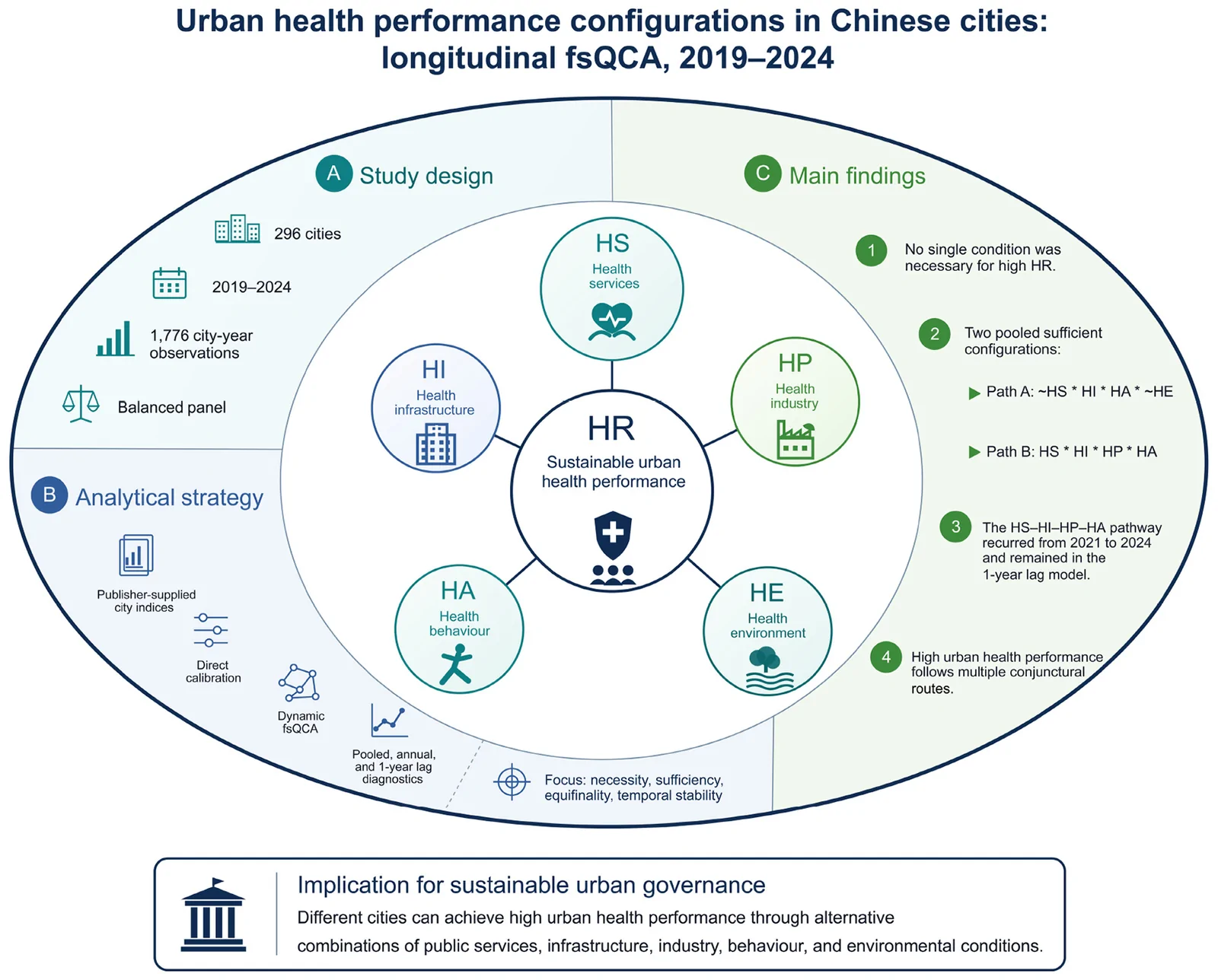

## 1 Introduction

Cities concentrate development opportunities, environmental pressures, and social inequalities. Population health is therefore both an outcome of urban development and a condition that enables cities to remain productive, inclusive, and resilient. The 2030 Agenda connects good health and wellbeing with sustainable cities and communities, while the healthy-cities tradition emphasizes that health is shaped well-beyond the health-care sector. Urban services, physical infrastructure, economic organization, everyday behavior, and environmental quality operate jointly, and comparable health performance may arise from different combinations of these domains (1, 2). A sustainability-oriented analysis must therefore move beyond isolated determinants and examine how social, economic, governance, behavioral, and environmental conditions interact.

China's Healthy China agenda has intensified city-level monitoring and cross-sector governance (3–5). Substantive urban-health research shows that rapid urbanization simultaneously reshapes health-care access, infectious and chronic disease burdens, health behavior, and environmental exposure (6). Spatial access to medical facilities and primary care remains markedly unequal across Chinese regions and cities (7, 8), while multicity evidence links particulate air pollution with daily mortality and broader work documents the continuing health burden of degraded air and water (9, 10). These findings make services, infrastructure, behavior, and environment substantively relevant rather than merely convenient index domains. Composite indices and spatial assessments facilitate multidimensional comparison (11), but rankings and average-effect models do not show whether favorable performance is associated with one dominant domain, coordinated membership across domains, or multiple substitutable conjunctions. Existing configurational urban studies have focused mainly on general sustainability or green performance (12–14), leaving unresolved which urban-health configurations recur over time and whether the cities displaying them remain the same.

Fuzzy-set qualitative comparative analysis (fsQCA) is suited to this question because it evaluates necessity, sufficiency, conjunctural relations, equifinality, and asymmetry rather than estimating the average net effect of each condition (15–17). QCA has been applied to complex public-health and implementation settings in which outcomes are associated with combinations of interventions and contexts (18–20). A conventional cross-sectional fsQCA cannot reveal whether a sufficient configuration recurs across years, and a regression coefficient cannot identify multiple logically substitutable set expressions. Conversely, a pooled solution may conceal temporal instability, whereas separate annual solutions may overlook whether the same cities sustain membership. Temporal QCA research therefore recommends repeated solutions, lag structures, and case-based sequence diagnostics (21–23).

The analytical framework is inductively derived from the TUHI hierarchy rather than presented as an independently validated causal theory. It is nevertheless aligned with whole-system healthy-cities thinking, which treats population health as jointly embedded in public provision, the built environment, economic organization, resident practices, and ecological conditions (1, 2, 6). Within this source-grounded framework, HS and HI represent public service and spatial provision, HP represents industrial and market capacity, HA represents resident practices, and HE represents environmental context. HR is the separate health-outcome domain. We ask whether any single condition is necessary; which configurations are sufficient for high and non-high HR; how stable the configurations and city memberships are across years; and whether the principal relations persist under lagging, calibration changes, sample perturbations, component removal, and subgroup diagnostics. The contribution is to replace a single ranking or average-effect account with a longitudinal, set-theoretic description of recurring and heterogeneous urban-health configurations, while explicitly bounding interpretation to associations within the TUHI measurement system.

## 2 Materials and methods

### 2.1 Study design and data source

This study draws on the Tsinghua Urban Health Index (TUHI), a city-level assessment system developed under the guidance of the National Health Commission by the Institute for China Sustainable Urbanization and the Vanke School of Public Health at Tsinghua University. The framework translates the broad “health + city” concept into a China-adapted measurement system using six first-level domains, 16 second-level components, and 39 third-level indicators. The public release provides annual first-level scores for 296 prefecture-level and above cities; the manuscript now distinguishes these source-provided scores from the unavailable raw records and aggregation files.

TUHI combines conventional official statistics with multi-source social big data. The public documentation identifies statistical yearbooks, departmental surveys, and census records as official inputs, and describes large-scale application, payment, enterprise, search-engine, and online-map data for behavior, consumption, industry, attention, and facility accessibility. The authors received only the released six-domain workbook and did not access personally identifiable records, provider-level microdata, or the raw normalization and weighting files. Consequently, the analysis audits the released panel but cannot independently verify every upstream data-collection or fusion procedure.

The workbook covered publication years 2020–2025. Following the portal's dating convention, each publication year was mapped to the preceding analytical year, yielding 2019–2024 while retaining publication year in the audit file. Administrative-code harmonization confirmed the same 296 cities in every year. The final balanced panel therefore comprised 1,776 observations (296 cities × 6 years); no city or year was excluded, and the six analysis variables contained no duplicate city-year records or missing values. Variable labels, units, and displayed precision were consistent across releases.

The design is observational and ecological, with the city-year as the unit of analysis. The 1,776 records are not treated as statistically independent replications. Large-N fsQCA remains appropriate because the estimand is a set-theoretic sufficiency relation rather than a sampling-based regression coefficient; however, repeated measurements can make a pooled relation appear more stable than it is. We therefore used common pooled calibration anchors, annual solutions, pooled/between-year/within-city cluster diagnostics, dominant-configuration transitions, a 1-year lag specification, and city-clustered train-test splits in which all observations from a city remained together. These diagnostics address temporal dependence descriptively but do not create an independent causal design. City size and the four macro-regions were used only for stratified diagnostics.

### 2.2 Measurement framework, outcome, and conditions

The TUHI structure aggregates 39 third-level indicators into 16 second-level components and then into six first-level domain scores. The outcome was urban health utility (HR), the English label used here for the source's health-outcome domain. HR combines infectious-disease control, chronic-disease management, and physical fitness; specifically, its three underlying indicators concern notifiable infectious-disease incidence, premature mortality from major chronic diseases, and the proportion meeting the National Physical Fitness Measurement Standards. HR is therefore a composite city-level health-performance score, not preference-based health utility, quality-adjusted life-years, an individual clinical outcome, or the overall TUHI. It is used as a bounded proxy for urban health-related sustainability because it captures whether cities simultaneously perform on communicable disease, chronic disease, and physical fitness.

The five conditions are HS, HI, HP, HA, and HE. The published hierarchy assigns each of the 39 third-level indicators to one and only one first-level domain: 36 distinct indicators feed the five condition domains, while three different indicators feed HR. Thus, no condition score is arithmetically included in HR and no third-level indicator is shared between a condition and the outcome in the published hierarchy (Table 1). Nevertheless, all six scores originate from the same TUHI provider and share an undisclosed normalization, weighting, and aggregation environment. The absence of item overlap reduces direct mathematical circularity but does not eliminate common-method dependence or correlations induced by shared scaling. Because the raw weights and aggregation files are unavailable, the study cannot independently reproduce the six domain scores; all findings are therefore interpreted as associations among released domains within the TUHI system rather than as external causal validation of independent constructs.

**Table 1:** Indicator hierarchy underlying the six source-provided TUHI domain scores.

First-level domain	Second-level component	Underlying third-level indicators
**HS—Health services**	HS1 Medical and health resources	Hospital beds per 1,000 population; licensed (assistant) physicians per 1,000 population
	HS2 Financial protection	Per capita government health expenditure; actual reimbursement ratio for inpatient expenses under basic medical insurance
**HI—Health infrastructure**	HI1 Core facilities	Per capita medical and health facilities; care facilities for older adults; parks and plazas; sports and fitness facilities
	HI2 Supporting facilities	Per capita traditional Chinese medicine facilities; psychological service facilities; public toilets; public transit stops; emergency shelters
	HI3 Layout and operation	Accessibility of community health facilities; completeness of community health facilities
**HP—Health industry**	HP1 Industry scale	Number of market entities; registered-capital scale; capital-attraction capacity
	HP2 Industry advantage	Share of listed companies; share of specialized, refined, distinctive, and innovative SMEs; industrial agglomeration
	HP3 Innovation capacity	Patent R&D level; new-drug R&D level; standard-setting capacity
**HA—Health behavior**	HA1 Exercise habits	Average outdoor exercise duration; average outdoor exercise distance
	HA2 Health expenditure	Per capita medical and healthcare consumption; per capita sports-goods consumption
	HA3 Health awareness	Attention to physical health issues; attention to mental health issues; residents' health literacy
**HE—Health environment**	HE1 Vegetation and greening	Per capita park green space; green coverage rate of built-up areas; remote-sensing vegetation index
	HE2 Air quality	Air Quality Index (AQI); number of heavily polluted days
**HR—Urban health utility**	HR1 Infectious-disease control	Incidence of Class A and Class B notifiable infectious diseases
	HR2 Chronic-disease management	Premature mortality from major chronic diseases
	HR3 Physical fitness	Proportion of urban and rural residents achieving qualified-or-higher scores under the National Physical Fitness Measurement Standards

The published hierarchy assigns every third-level indicator to a single first-level domain. The five condition domains contain 36 distinct indicators and HR contains three different outcome indicators; no listed third-level indicator overlaps between a condition and HR. Shared data provenance and undisclosed aggregation procedures remain potential sources of common-method dependence.

### 2.3 Analytical use of the published domain scores

Table 1 documents construct content and the non-overlapping assignment of the published third-level indicators; it does not imply access to or re-estimation of those indicators. All six first-level scores were analyzed exactly as released. No city-year value was reconstructed, rescaled, imputed, winsorized, deleted, or manually adjusted. The analysis consequently evaluates configurational associations within the original TUHI measurement system, and the conclusions do not claim that HR was independently measured outside that system.

### 2.4 Calibration

Descriptive statistics for the six source-provided indices are presented in Table 2, and the corresponding calibration anchors are shown in Table 3. Each index was transformed into fuzzy-set membership using direct logistic calibration. The main specification used pooled P5, P50, and P95 values as full non-membership, crossover, and full membership anchors, respectively, with an inclusion degree of 0.95. External policy thresholds were not available in comparable units for all six proprietary domain scores, so empirical anchors were necessary. P5/P95 were selected to distinguish the distributional tails while limiting leverage from extreme observed values, and the pooled median supplied a common point of maximum ambiguity. Pooling anchors across all years kept set membership comparable over time. Twelve variable-case combinations fell exactly at 0.5; these values were retained and were not shifted by an arbitrary constant. Sensitivity analyses repeated the models with pooled P10/P50/P90 and P25/P50/P75 anchors, thereby testing whether the main configurations depended on the chosen tail cut-offs.

**Table 2:** Descriptive statistics for the six source-provided urban health indices.

Code	Construct	*N*	Mean	SD	Min	P25	Median	P75	Max
HS	Government health services	1,776	62.36	7.01	46.00	57.10	61.99	67.15	99.24
HI	Health infrastructure	1,776	63.44	5.90	48.55	59.40	62.67	66.81	91.01
HP	Health industry	1,776	60.48	3.34	51.10	59.44	60.15	61.39	84.67
HA	Health behavior	1,776	62.76	4.75	52.91	59.28	61.96	65.43	88.56
HE	Health environment	1,776	64.04	9.45	41.37	56.49	65.53	71.47	92.59
HR	Urban health utility	1,776	60.78	9.79	24.95	55.29	60.62	66.19	145.44

**Table 3:** Direct-calibration anchors for the main analysis.

Code	Construct	Full non-membership	Crossover	Full membership
HS	Government health services	51.782	61.995	74.013
HI	Health infrastructure	55.237	62.675	74.010
HP	Health industry	54.220	60.150	65.782
HA	Health behavior	56.498	61.960	71.573
HE	Health environment	48.060	65.530	77.790
HR	Urban health utility	45.630	60.620	74.720

Main anchors are pooled P5, P50, and P95 values; direct logistic calibration used an inclusion degree of 0.95. Twelve exact crossover memberships of 0.5 were retained. Alternative P10/P50/P90 and P25/P50/P75 anchors were assessed in the robustness grid.

### 2.5 Necessity and pooled sufficiency analysis

For high HR and non-high HR (~HR), every condition and its negation was assessed as potentially necessary. A consistency of 0.90 served as the prespecified necessity reference and was interpreted jointly with coverage and relevance of necessity (RoN) (17, 24). For pooled sufficiency, the truth table used consistency of 0.85, proportional reduction in inconsistency (PRI) of 0.70, and a case-frequency threshold of 10. Complex, parsimonious, and intermediate solutions were estimated, and the first intermediate solution is reported. Directional expectations favored membership in all five conditions for high HR and non-membership for ~HR. If no row met all prespecified thresholds, the result was recorded as no solution rather than obtained by *post-hoc* threshold relaxation.

### 2.6 Longitudinal diagnostics

We evaluated the pooled high-HR solution using cluster diagnostics that distinguish pooled, between-year, and within-city consistency and coverage (22, 25). Annual QCA models retained the same consistency and PRI thresholds and used a frequency threshold of 5. Year-specific configurations were linked into families when their signed-condition Jaccard similarity with a configuration in the preceding observed year was at least 0.50. For each city-year, the family with the greatest configuration membership was designated dominant when membership reached at least 0.50. These layers prevent the repeated city observations from being interpreted as independent evidence of temporal stability.

To assess temporal ordering, all five conditions were lagged by 1 year, linking conditions in 2019–2023 to HR in 2020–2024 (*N* = 1,480). The lagged model used the same pooled truth-table thresholds. Lagging does not eliminate time-varying confounding, identify a counterfactual, or establish a mechanism; it is interpreted only as a temporal robustness check on set-theoretic associations.

### 2.7 Robustness, component, and subgroup analyses

Following guidance on robustness in large-N QCA (26), we estimated a parameter grid combining consistency thresholds of 0.80, 0.85, and 0.90; PRI thresholds of 0.65, 0.70, and 0.75; frequencies of 5, 10, 15, and 20; and three pooled calibration schemes (95/50/5, 90/50/10, and 75/50/25). This produced 108 high-HR models. We also estimated 40 sample variations, five leave-one-condition-out models, and 100 city-clustered 70/30 train-test splits. All observations from a city remained in the same split, and held-out consistency, PRI, and coverage were calculated from each training solution.

Subgroup QCA was conducted separately by annual city-size category and by macro-region. City-size models used a frequency threshold of 5, whereas regional models used a threshold of 3. Nine cities changed size category during the study period and therefore contributed observations to both size strata. Because several subgroup solutions fell below the main consistency threshold and regional samples were smaller, these analyses are treated as exploratory heterogeneity diagnostics.

### 2.8 Software and reproducibility

Data management and QCA were performed in R 4.4.2 using QCA 3.23 and SetMethods 4.1 (27, 28).

## 3 Results

### 3.1 No single condition was necessary

No positive or negated condition reached the prespecified necessity-consistency reference of 0.90 for high HR (Table 4 and Figure 1). HP had the highest necessity consistency (0.756), followed by HI (0.734) and HA (0.731). Coverage was moderate, and RoN ranged from 0.694 to 0.820. Thus, across the pooled panel, high urban health performance was not constrained by membership or non-membership in any single condition.

**Table 4:** Necessity analysis for high urban health performance.

Condition	Construct	Consistency	Coverage	RoN	≥0.90
HS	Government health services	0.695	0.712	0.781	No
HI	Health infrastructure	0.734	0.760	0.814	No
HP	Health industry	0.756	0.728	0.769	No
HA	Health behavior	0.731	0.765	0.820	No
HE	Health environment	0.614	0.624	0.729	No
~HS	Government health services	0.616	0.614	0.716	No
~HI	Health infrastructure	0.599	0.591	0.699	No
~HP	Health industry	0.644	0.684	0.777	No
~HA	Health behavior	0.604	0.589	0.694	No
~HE	Health environment	0.669	0.672	0.751	No

The tilde (~) denotes non-membership. RoN denotes relevance of necessity. HP had the highest necessity consistency (0.756), which remained below the 0.90 reference.

**Figure 1:**
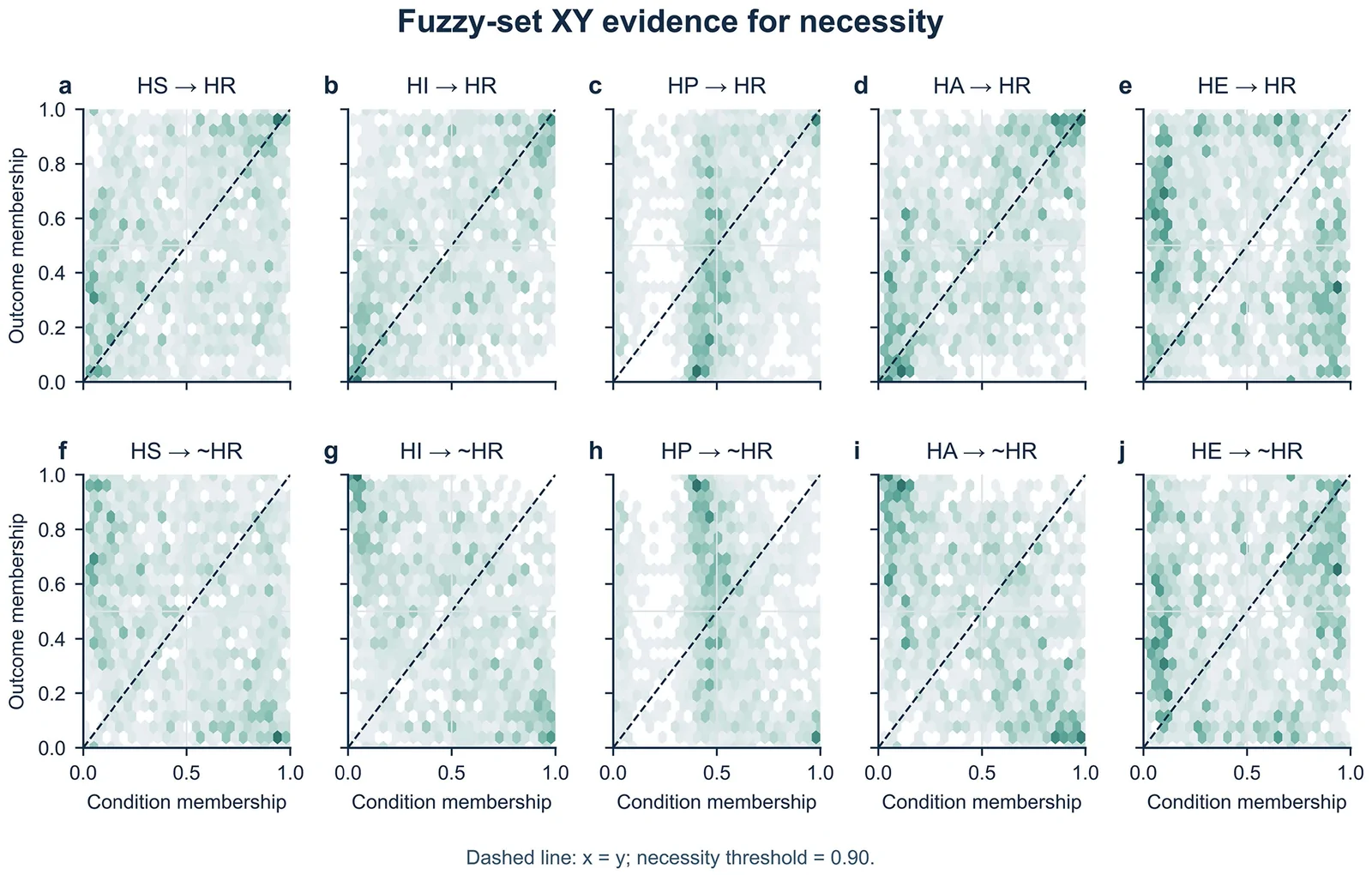
Fuzzy-set XY evidence for necessity. [Panels **(a–e)**] plot each condition set against high urban health utility (HR), and [panels **(f–j)**] plot the same conditions against non-high urban health utility (~HR). Hexagonal bins summarize 1,776 city-year observations. The dashed diagonal represents *x* = *y*; systematic observations above the line would support necessity. No condition reached the prespecified necessity-consistency reference of 0.90.

### 3.2 Two equifinal configurations were sufficient in the pooled panel

The pooled intermediate solution comprised two sufficient configurations (Table 5). Configuration 1 (~HS^*^HI^*^HA^*^~HE) combined high membership in infrastructure and health behavior with lower membership in government health services and health environment. Its consistency was 0.926, PRI was 0.725, and raw coverage was 0.329. This expression is a set association under relative calibration; it does not mean that lower HS or HE improves HR. Configuration 2 (HS^*^HI^*^HP^*^HA) combined high membership in services, infrastructure, industry, and health behavior, while HE was unconstrained. Its consistency was 0.877, PRI was 0.733, and raw coverage was 0.476.

**Table 5:** Pooled intermediate solution for high urban health performance.

Config.	Expression	Interpretive label	Consistency	PRI	Raw coverage	Unique coverage
C1	~HS^*^HI^*^HA^*^~HE	Infrastructure–behavior configuration under lower HS and HE membership	0.926	0.725	0.329	0.064
C2	HS^*^HI^*^HP^*^HA	Four-domain coordination configuration	0.877	0.733	0.476	0.211

C1 and C2 denote Configuration 1 and Configuration 2. Solution consistency = 0.872; solution PRI = 0.723; solution coverage = 0.540. The pooled truth table used consistency 0.85, PRI 0.70, and frequency 10. Under the same thresholds, ~ HR had no row coded as sufficient. A tilde denotes lower fuzzy-set membership, not literal absence or a beneficial effect of low membership. The asterisk (*) denotes logical AND (set conjunction) between conditions in a configuration.

The union of the two configurations had consistency of 0.872, PRI of 0.723, and coverage of 0.540. Configuration 2 provided greater unique coverage than Configuration 1 (0.211 vs. 0.064), so it covered a larger distinct share of high-HR membership. Complex, parsimonious, and intermediate minimization returned the same expressions. Under the same thresholds, the pooled ~HR analysis contained no positive truth-table row. This threshold-bounded no-solution result demonstrates configurational asymmetry; it does not imply that non-high HR lacks explanations.

### 3.3 Between-year and within-city diagnostics showed unequal stability

Between-year consistency for Configuration 1 exceeded 0.85 in every year, rising from 0.878 in 2019 to 0.970 in 2024 (Figure 2). Configuration 2 fell below 0.85 in 2019 (0.810) and 2020 (0.822), but ranged from 0.882 to 0.902 during 2021–2024. The union similarly remained below 0.85 in 2019 and 2020 and exceeded the threshold from 2021 onwards. The pooled relation was therefore supported more clearly in the later releases.

**Figure 2:**
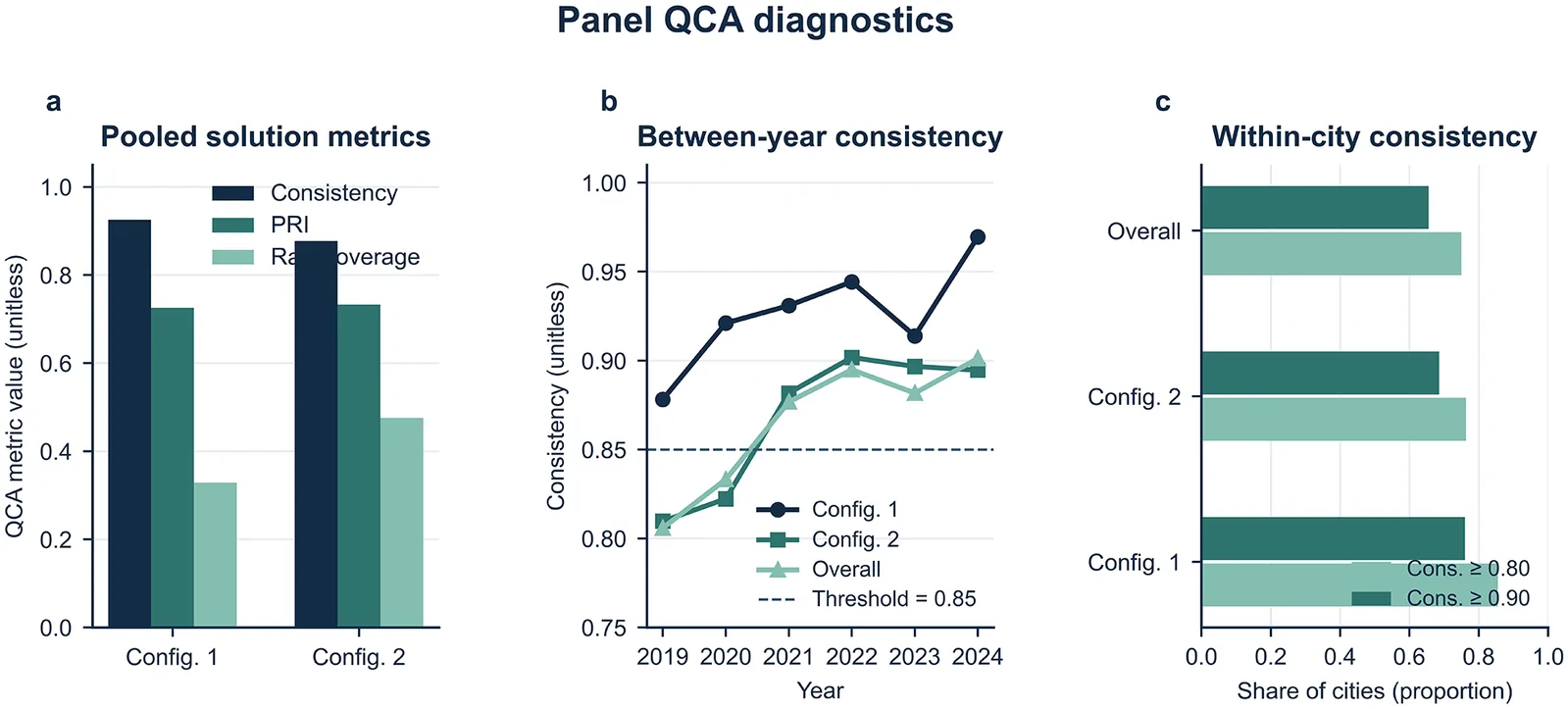
Pooled, between-year, and within-city QCA diagnostics. **(a)** Pooled consistency, proportional reduction in inconsistency (PRI), and raw coverage for the two sufficient configurations. **(b)** Between-year consistency for Configuration 1 (~HS*HI*HA*~HE), Configuration 2 (HS*HI*HP*HA), and their union; the dashed line marks the 0.85 sufficiency threshold. c Shares of cities with within-city consistency of at least 0.80 or 0.90.

Within-city consistency was at least 0.80 for 85.5% of cities for Configuration 1, 76.4% for Configuration 2, and 75.0% for their union. At 0.90, the corresponding proportions were 76.0%, 68.6%, and 65.5%. Only 17 cities retained the same dominant pooled configuration across all 6 years; 124 switched between dominant families, whereas 155 did not maintain membership of at least 0.50 in a main family throughout the panel. Pooled sufficiency therefore coexisted with substantial city-level heterogeneity.

### 3.4 Annual solutions changed, but one configuration recurred

At the annual thresholds, no high-HR solution was obtained for 2019, one configuration was identified for 2020, and two were identified in each year from 2021 to 2024 (Table 6; Figures 3, 4). The 2019 result is traceable to the truth table: no row simultaneously met consistency 0.85, PRI 0.70, and frequency 5. It was not produced by missingness, duplicate records, or city exclusion because all 296 cities were present and complete. We did not lower a threshold *post-hoc*. Because the available technical files do not permit independent verification of upstream aggregation changes, we cannot determine whether measurement or the pre-pandemic policy context contributed; the result should therefore be read as a threshold-bounded annual difference, not as evidence of a COVID-19 effect. The HS^*^HI^*^HP^*^HA configuration appeared in every annual model from 2021 through 2024; accompanying expressions combined HI, HP, and HA with ~HE in 2021 and 2023, HA with ~HE in 2022, and HS and HI with ~HE in 2024.

**Table 6:** Year-specific intermediate solutions.

Year	Outcome	Expression(s)	Solution consistency	Solution PRI	Solution coverage	No.
2019	HR	No solution	–	–	–	0
2019	~HR	~HS^*^~HI^*^~HA^*^~HE + HS^*^~HI^*^~HP^*^~HA	0.826	0.694	0.595	2
2020	HR	~HS^*^HI^*^HP^*^HA^*^~HE	0.922	0.718	0.340	1
2020	~HR	~HS^*^~HI^*^~HA^*^HE	0.852	0.711	0.518	1
2021	HR	HS^*^HI^*^HP^*^HA + HI^*^HP^*^HA^*^~HE	0.877	0.700	0.530	2
2021	~HR	~HI^*^~HA^*^HE	0.878	0.755	0.546	1
2022	HR	HA^*^~HE + HS^*^HI^*^HP^*^HA	0.853	0.716	0.705	2
2022	~HR	No solution	–	–	–	0
2023	HR	HS^*^HI^*^HP^*^HA + HI^*^HP^*^HA^*^~HE	0.885	0.766	0.652	2
2023	~HR	No solution	–	–	–	0
2024	HR	HS^*^HI^*^~HE + HS^*^HI^*^HP^*^HA	0.900	0.814	0.623	2
2024	~HR	No solution	–	–	–	0

Annual models used consistency 0.85, PRI 0.70, and frequency 5. No 2019 high-HR truth-table row met all three thresholds; all 296 city records were complete. The asterisk (*) denotes logical AND (set conjunction) between conditions in a configuration.

**Figure 3:**
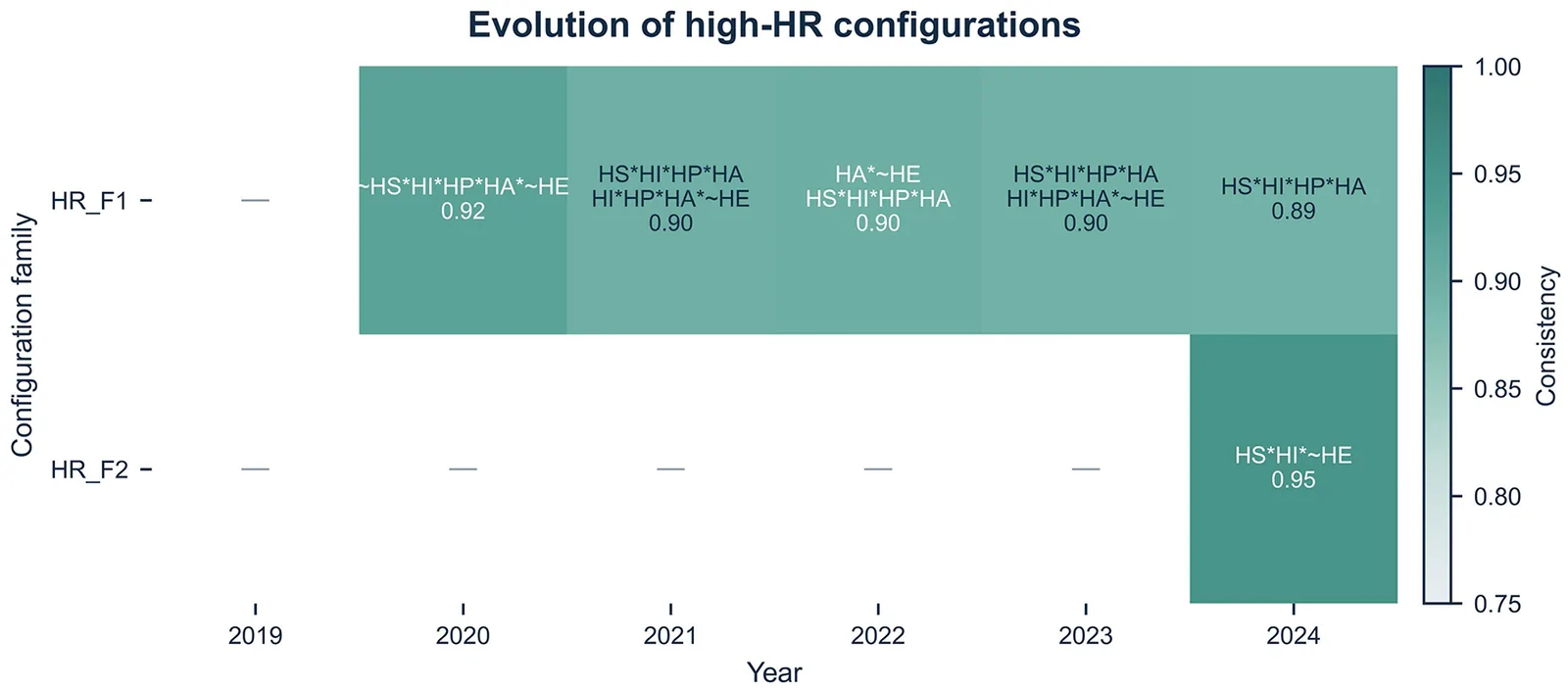
Evolution of sufficient configurations for high urban health performance. Year-specific intermediate solutions are grouped into cross-year families using signed-condition Jaccard similarity of at least 0.50 relative to the preceding observed year. Cell labels report the observed expression or expressions and configuration consistency. Blank cells indicate that the family was not identified in that year.

**Figure 4:**
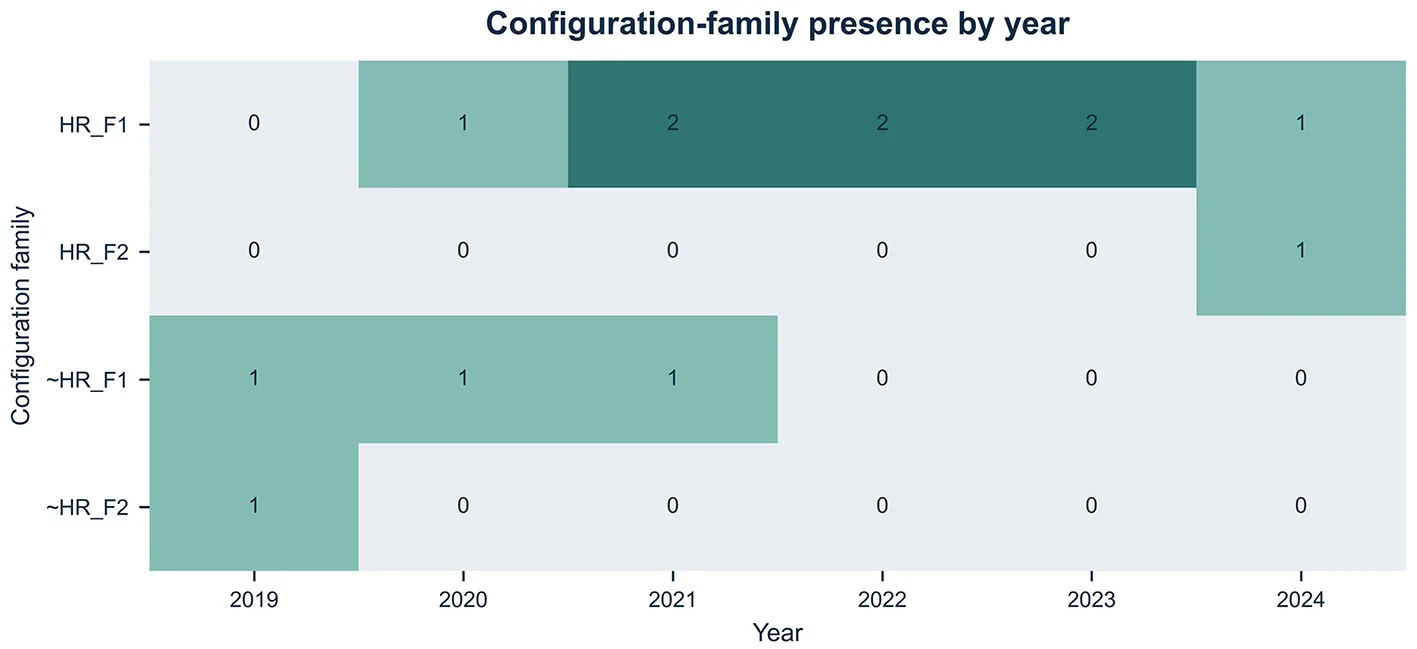
Appearance and disappearance of configuration families.

The ~HR analysis followed a different sequence: two configurations appeared in 2019, one in 2020, and one in 2021, whereas no ~HR solution was obtained from 2022 to 2024. This sequence records the occurrence of sufficient set relations within the observed panel and does not establish strict year-to-year causal change in population health.

### 3.5 Dominant city configurations changed over time

Among consecutive years for which high-HR families could be assigned, 67% of city-years dominated by HR_F1 remained in HR_F1 in the following year, 30% moved below the 0.50 dominant-configuration threshold, and 4% shifted to HR_F2 (Figure 5). Of city-years without a dominant configuration, 80% remained without one, 18% entered HR_F1, and 2% entered HR_F2. These are transitions in fuzzy-set configuration membership, not population migration, commuting, or inter-city movement.

**Figure 5:**
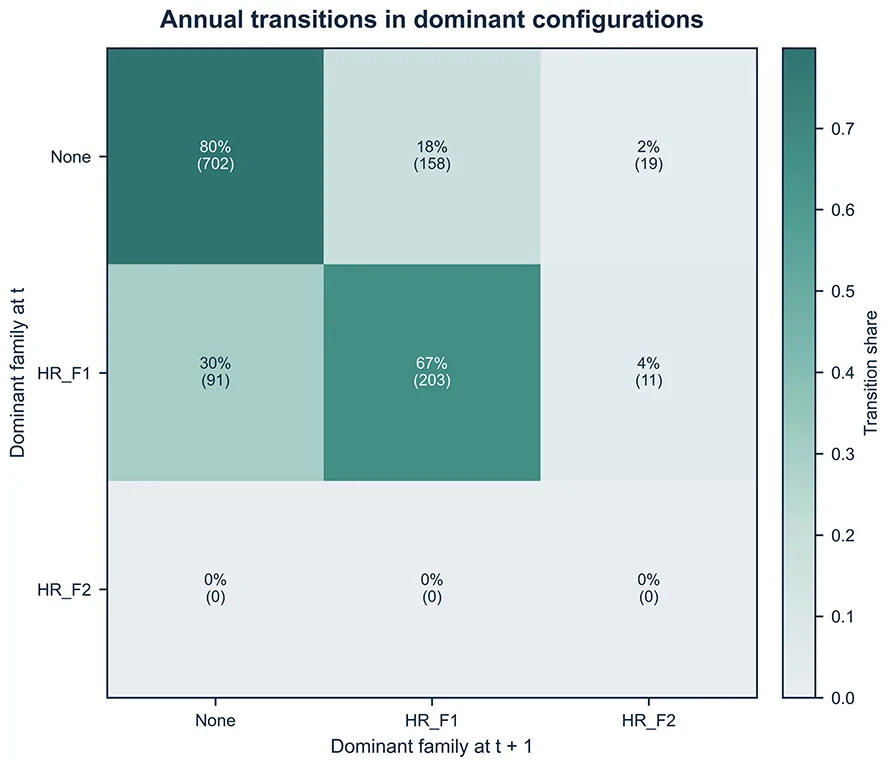
Annual transitions in city-level dominant configuration families. For each city-year with an available high-HR annual solution, the family with the largest membership was designated dominant when membership was at least 0.50; otherwise, the observation was classified as having no dominant configuration. Cells report row-normalized transition percentages and counts across consecutive available years. HR_F1 is the recurrent high-HR family; HR_F2 first appeared in 2024 and therefore has no outgoing transition in the observed sequence.

### 3.6 The main four-domain configuration persisted in the lagged model

The 1-year lagged analysis produced two configurations: HA^*^~HE and HS^*^HI^*^HP^*^HA (Table 7). The first had consistency of 0.862, PRI of 0.709, and raw coverage of 0.507; the second had consistency of 0.900, PRI of 0.778, and raw coverage of 0.471. Their union achieved consistency of 0.853, PRI of 0.712, and coverage of 0.596. Relative to the contemporaneous pooled solution, the first lagged expression simplified to HA^*^~HE (signed-condition Jaccard similarity = 0.50), whereas the four-domain configuration was reproduced unchanged (similarity = 1.00). These are temporally ordered associations, not lagged causal effects.

**Table 7:** One-year lagged intermediate solution for high urban health performance.

Expression	Interpretive label	Consistency	PRI	Raw coverage	Unique coverage
HA^*^~HE	Behavior–environment configuration	0.862	0.709	0.507	0.125
HS^*^HI^*^HP^*^HA	Four-domain coordination configuration	0.900	0.778	0.471	0.089

N = 1,480 city-year observations, linking conditions in 2019–2023 to HR in 2020–2024. Solution consistency = 0.853; solution PRI = 0.712; solution coverage = 0.596. Signed-condition Jaccard similarity with the contemporaneous pooled configurations was 0.50 for the first expression and 1.00 for the four-domain expression. The asterisk (*) denotes logical AND (set conjunction) between conditions in a configuration.

### 3.7 Robustness was strongest under the main calibration

All 36 high-HR parameter models under the main 95/50/5 calibration produced a solution, with mean main-configuration retention of 0.870 (Table 8 and Figure 6). Under 90/50/10 calibration, 16 of 36 models produced a solution and mean retention among defined results was 1.000. Under 75/50/25 calibration, only 8 of 36 models produced a solution and mean retention was 0.500. Thus, the main configurations were reproducible across truth-table thresholds under the principal calibration, but solution recovery weakened when the calibration anchors were compressed.

**Table 8:** Robustness and held-out validation summary.

Analysis	Models with solution	Mean config. retention	Mean consistency	Mean PRI	Mean coverage
Calibration 95/50/5	36/36	0.870	0.882	0.726	0.480
Calibration 90/50/10	16/36	1.000	0.855	0.716	0.353
Calibration 75/50/25	8/36	0.500	0.779	0.710	0.253
Forty sample variations	40/40	0.992	0.869	0.720	0.545
City-clustered train-test	99/100	–	0.866	0.609	0.532

For calibration grids, means are calculated across models that produced a solution and for which the metric was defined. Main-configuration retention is the proportion of recovered configurations with signed-condition Jaccard similarity of at least 0.50 to a pooled main configuration. The 100 train-test repetitions split cities, not city-years, into 70% training and 30% testing sets.

**Figure 6:**
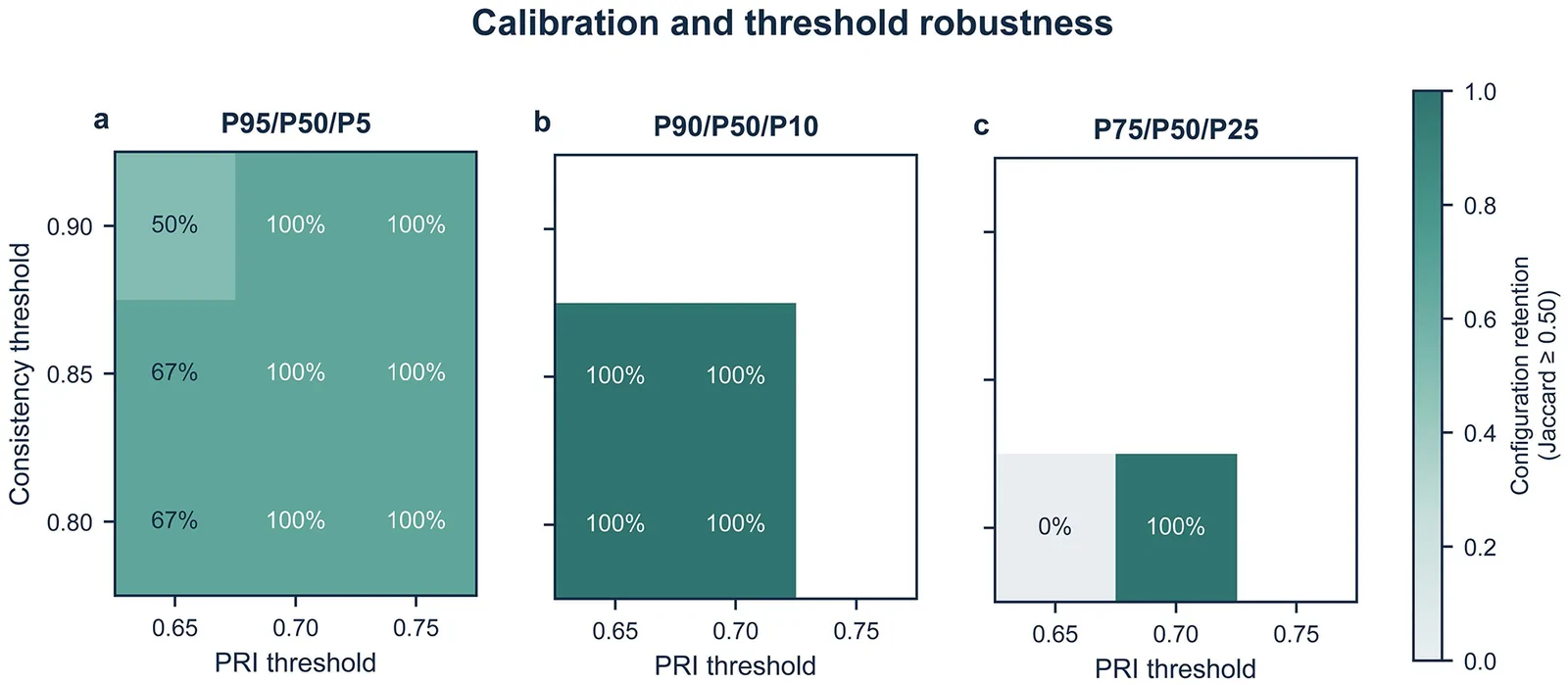
Robustness to calibration anchors and sufficiency thresholds. Panels show main-configuration retention, defined by signed-condition Jaccard similarity of at least 0.50, across combinations of consistency and PRI thresholds. Frequencies of 5, 10, 15, and 20 are aggregated within each cell. Empty cells denote parameter combinations with no high-HR solution. The calibration schemes use pooled percentiles **(a)** 95/50/5, **(b)** 90/50/10, and **(c)** 75/50/25 for full membership, crossover, and full non-membership, respectively.

All 40 sample-variation models produced a solution, with mean configuration retention of 0.992. In the city-clustered train-test analysis, 99 of 100 training samples yielded a solution. Across valid repetitions, mean held-out consistency was 0.866 (10th−90th percentiles, 0.834–0.895), mean held-out coverage was 0.532, and mean held-out PRI was 0.609. The lower held-out PRI indicates non-trivial contradictory membership in unseen cities, even though average consistency remained above 0.85. Subgroup results also differed by city size and region; the robustness analyses therefore support the main patterns while identifying meaningful calibration and contextual limits.

### 3.8 Robustness was strongest under the main calibration

Each leave-one-condition-out model produced an alternative high-HR solution (Table 9 and Figure 7). Solution consistency ranged from 0.877 after excluding HE to 0.898 after excluding HS, whereas coverage ranged from 0.389 after excluding HA to 0.476 after excluding HE. No positive condition assessed alone reached sufficiency consistency of 0.90. Removing a condition therefore changed the minimized configuration without eliminating all sufficient expressions; this is evidence of configurational substitutability, not of a causal compensatory mechanism or an unimportant excluded domain.

**Table 9:** Leave-one-condition-out component analysis.

Excluded condition	Alternative expression	Consistency	PRI	Coverage	Max Jaccard
HS	HI^*^HP^*^HA^*^~HE	0.898	0.738	0.414	0.600
HI	HS^*^HP^*^HA^*^~HE	0.896	0.731	0.390	0.600
HP	HI^*^HA^*^~HE	0.881	0.718	0.462	0.750
HA	HS^*^HI^*^HP^*^~HE	0.890	0.715	0.389	0.600
HE	HS^*^HI^*^HP^*^HA	0.877	0.733	0.476	1.000

All five reduced models produced an alternative high-HR solution at the main thresholds. This indicates configurational substitutability within the analyzed condition set and should not be read as evidence that an excluded condition has no substantive importance. The asterisk (*) denotes logical AND (set conjunction) between conditions in a configuration.

**Figure 7:**
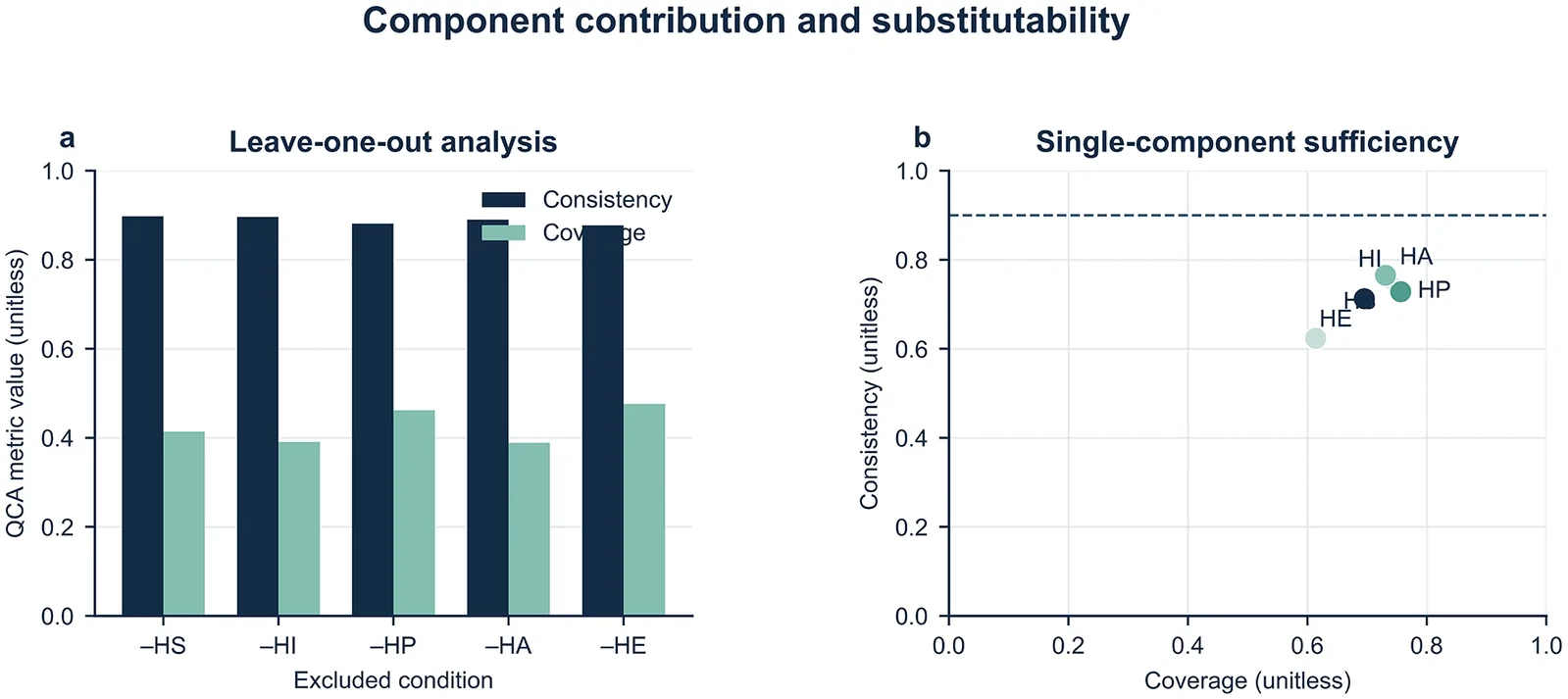
Component contribution and configurational substitutability. **(a)** Solution consistency and coverage after excluding each condition from the five-condition model in turn. **(b)** Consistency and coverage when each positive condition is assessed alone as sufficient for high urban health performance; the dashed line marks 0.90. Every leave-one-condition-out model yielded an alternative solution, whereas no single positive condition was sufficient at 0.90.

### 3.9 Subgroup solutions were heterogeneous and mostly exploratory

Small and medium-sized cities yielded no high-HR solution but one ~HR configuration with solution consistency of 0.873 and coverage of 0.446. Large cities yielded two high-HR configurations, although their union had consistency of only 0.755 despite coverage of 0.846. Regional analyses identified high-HR solutions in the Northeast, East, and Central regions and ~HR solutions in the West. Only the Central high-HR solution exceeded overall consistency of 0.85 (0.948), and its coverage was 0.367. The remaining regional unions had consistency values between 0.775 and 0.834 (Table 10 and Figure 8).

**Table 10:** Within-group QCA by city size and region.

Group	Outcome	Observations	Cities	Frequency	No.	Consistency	Coverage	Status
Small/medium cities	HR	1,256	213	5	0	–	–	No solution
Small/medium cities	~HR	1,256	213	5	1	0.873	0.446	Solution
Large cities	HR	520	92	5	2	0.755	0.846	Solution
Large cities	~HR	520	92	5	0	–	–	No solution
Northeast	HR	204	34	3	3	0.834	0.704	Solution
Northeast	~HR	204	34	3	0	–	–	No solution
East	HR	522	87	3	4	0.775	0.910	Solution
East	~HR	522	87	3	0	–	–	No solution
Central	HR	480	80	3	1	0.948	0.367	Solution
Central	~HR	480	80	3	0	–	–	No solution
West	HR	570	95	3	0	–	–	No solution
West	~HR	570	95	3	4	0.804	0.826	Solution

Nine cities changed annual size category, so the unique-city counts for the two size strata overlap. The large-city, Northeast, East, and West solutions had overall consistency below 0.85 and are interpreted as exploratory heterogeneity diagnostics.

**Figure 8:**
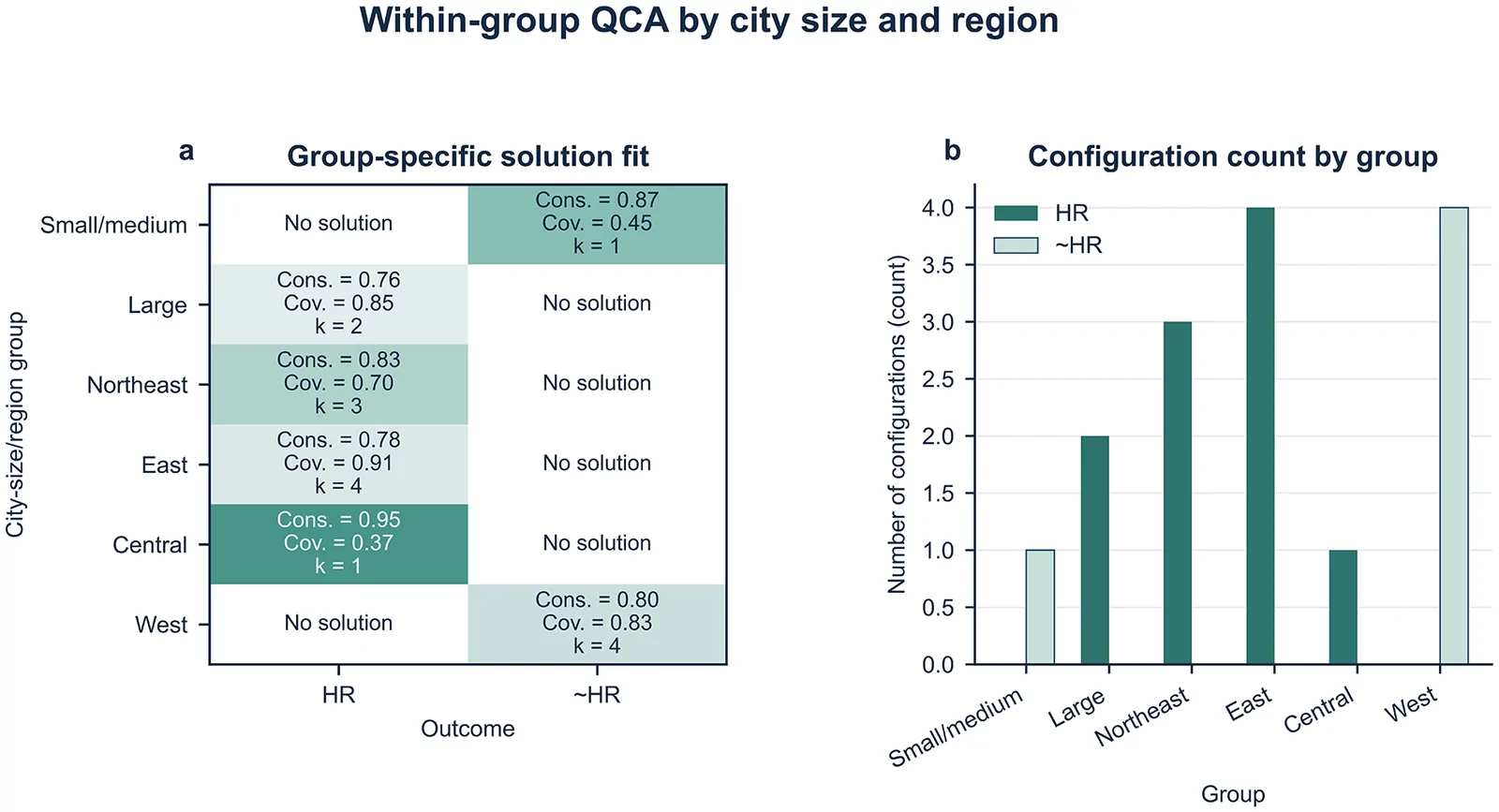
Within-group QCA by city size and region. **(a)** Solution consistency, coverage, and number of intermediate configurations for high HR and ~HR within city-size and regional strata. **(b)** Number of configurations by group and outcome direction. Annual city-size classifications were retained, so nine cities contributed observations to both size strata across the six-year panel. Regional models used a frequency threshold of 3 and city-size models a threshold of 5. Subgroup solutions with overall consistency below 0.85 are exploratory.

These subgroup results are exploratory heterogeneity diagnostics rather than separate confirmatory models. They show that the pooled configurations do not represent all city-size and regional contexts equally well, but they do not justify causal comparisons or strong rankings of regions and city types.

## 4 Discussion

### 4.1 Principal findings and contribution to urban sustainability

This study identified two equifinal configurations associated with high health-related urban sustainability performance across 296 Chinese cities. Neither reduced to a single necessary or sufficient input. The more broadly covering configuration combined government health services, health infrastructure, health industry, and health behavior; the alternative combined infrastructure and behavior with lower service and environmental membership. These expressions do not estimate causal effects. Their contribution is twofold: they move beyond composite rankings and subsystem averages by identifying sufficient conjunctions, and they add pooled, annual, between-year, within-city, transition, and lagged diagnostics to urban configurational studies that have largely used cross-sectional samples or non-health sustainability outcomes (12–14).

The four-domain coordination configuration is the most recurrent empirical pattern. It appeared in every annual model from 2021 to 2024, had the largest unique coverage in the pooled solution, and was reproduced unchanged in the lagged model. This recurrence is compatible with a whole-system interpretation in which favorable HR membership co-occurs with aligned services, infrastructure, industrial capacity, and resident practices. It does not demonstrate an independent positive effect for any domain, establish a mechanism called synergy, or imply that mechanically increasing all four scores will necessarily raise HR.

### 4.2 Temporal stability and dominant-configuration transitions

The annual and cluster diagnostics qualify the pooled findings. Between-year consistency strengthened after 2020, yet only a small minority of cities sustained the same dominant configuration throughout the panel. The transition matrix showed frequent changes between HR_F1 and the no-dominant-configuration state. A set relation can therefore recur at the pooled level while individual cities enter or leave membership in the configurations that sustain it.

This distinction matters for sustainability monitoring. An aggregate indicator can suggest a stable pattern while masking changing conjunctions of services, infrastructure, industry, behavior, and environment. The diagnostics record recurring and shifting fuzzy-set membership within a 6-year panel; they do not measure population migration, commuting, or an individual health trajectory, and they do not establish that a change in any one condition produced a later change in HR.

### 4.3 Sustainability interpretation and policy implications

The lower-HE configuration requires separate empirical and normative interpretation. Empirically, ~HE means that high HI and HA membership can co-occur with high HR membership when HE is below its pooled crossover; it does not show that environmental weakness improves health or is causally compensated by other domains. Normatively, weak sustainability permits substitution between natural and human-made capital, whereas strong sustainability treats critical natural capital and ecological thresholds as non-substitutable (29, 30). Because air quality and urban green systems directly shape health and can involve irreversible or unequally distributed harm, the observed configuration cannot justify trading environmental protection for performance elsewhere. A strong-sustainability reading therefore treats environmental standards as safeguards external to the empirical sufficiency claim.

Policy priorities should differ by observed configuration. For cities resembling Configuration 1 (~HS^*^HI^*^HA^*^~HE), the immediate task is to preserve infrastructure access and health-supporting behavior while closing service and environmental gaps: strengthen primary and preventive services, improve air-quality and green-space performance, and monitor whether HR remains high without persistent deficits. For cities resembling Configuration 2 (HS^*^HI^*^HP^*^HA), policy should consolidate coordination among service delivery, accessible facilities, responsible health-industry capacity, and resident participation, while adding an explicit environmental floor because HE is logically unconstrained in that expression. Cities with no dominant configuration require diagnosis before portfolio transfer. In all cases, these are priorities informed by observed associations, not predictions of causal effects.

### 4.4 Transferability, strengths, and limitations

The empirical configurations are not directly generalizable beyond the 296 Chinese cities because TUHI content, national institutions, urban administrative boundaries, and pooled calibration anchors are context-specific. The transferable element is the analytical strategy: other countries can define locally valid, non-overlapping condition and outcome sets and test their own pooled, annual, and within-unit relations. Cross-national transfer would require measurement invariance or explicit redefinition of constructs, locally meaningful calibration anchors, and replication under different governance and environmental regimes. Methodologically, the balanced panel, prespecified thresholds, separate HR and ~HR analyses, clustered diagnostics, lagging, parameter and sample perturbations, component removal, held-out validation, and subgroup analyses improve transparency but do not remove the contextual boundary.

Several limitations define the validity boundary. First, the 39 published third-level indicators are assigned without overlap across the six first-level domains, so HR does not arithmetically include HS, HI, HP, HA, or HE; nevertheless, all scores share a provider and an undisclosed normalization, weighting, and aggregation environment. Common-method dependence and scaling-induced correlations therefore remain possible, and the unavailable raw weights prevent independent reconstruction. Second, the released values were retained without imputation or deletion, but year-to-year measurement comparability could not be independently verified. This uncertainty also limits interpretation of the 2019 no-solution result, which may reflect the observed truth-table structure, upstream measurement, policy context, or their combination. Third, percentile calibration is empirical rather than tied to external policy thresholds, and compressed anchors reduced solution recovery. Fourth, 6 years are a short sequence; repeated observations were addressed with cluster, annual, transition, lag, and city-split diagnostics, but temporal dependence remains and lagging does not establish causality. Fifth, QCA reports set relations rather than net effect sizes. Sixth, several subgroup unions fell below 0.85 and mean held-out PRI was 0.609. Finally, omitted institutional, demographic, economic, environmental, and spatial conditions may support other configurations.

## 5 Conclusion

Across 296 Chinese cities, high HR membership was associated with two equifinal sufficient configurations and no single necessary condition. The HS^*^HI^*^HP^*^HA configuration was the most recurrent across pooled, annual, lagged, and sample analyses; policy for cities displaying it should consolidate coordinated services, infrastructure, responsible industry, and resident participation while enforcing an environmental floor. Cities displaying ~HS^*^HI^*^HA^*^~HE should preserve infrastructure and behavioral strengths but prioritize service and environmental deficits rather than regard lower membership as desirable. Cities without a dominant configuration require local diagnosis before transferring either portfolio. These conclusions are contextual, set-theoretic associations within the TUHI system. Longitudinal fsQCA complements city rankings and regression-based averages by showing both recurrent sufficient configurations and changes in city membership over time, without claiming causal effects.

## Data Availability

Publicly available datasets were analyzed in this study. This data can be found here: the source indices are publicly available from the Tsinghua University Urban Health Index portal (https://tuhi.tsinghua.edu.cn/en/managementPaper.html).
